# Genome-Wide Analysis and Expression Profiling of Rice Hybrid Proline-Rich Proteins in Response to Biotic and Abiotic Stresses, and Hormone Treatment

**DOI:** 10.3390/plants8090343

**Published:** 2019-09-11

**Authors:** Ritu Kapoor, Gulshan Kumar, Preeti Arya, Rajdeep Jaswal, Priyanka Jain, Kashmir Singh, Tilak Raj Sharma

**Affiliations:** 1 Department of Biotechnology, Panjab University, Chandigarh 160014, Punjab, India; ritukapoor1985@gmail.com; 2 National Agri-Food Biotechnology Institute, Mohali 140306, Punjab, India; gulshan.ihbt@gmail.com (G.K.); arya.preet9@gmail.com (P.A.); rajdeepjaswal52@gmail.com (R.J.); 3 Department of Microbiology, Panjab University, Chandigarh 160014, Punjab, India; 4 National Institute of Plant Biotechnology, New Delhi 110012, India; priybioinfo@gmail.com

**Keywords:** rice, hybrid proline-rich proteins, biotic stress, abiotic stress, *Magnaporthe oryzae*

## Abstract

Hybrid proline-rich proteins (HyPRPs) belong to the family of 8-cysteine motif (8CM) containing proteins that play important roles in plant development processes, and tolerance to biotic and abiotic stresses. To gain insight into the rice HyPRPs, we performed a systematic genome-wide analysis and identified 45 *OsHyPRP* genes encoding 46 OsHyPRP proteins. The phylogenetic relationships of OsHyPRP proteins with monocots (maize, sorghum, and *Brachypodium*) and a dicot (Arabidopsis) showed clustering of the majority of OsHyPRPs along with those from other monocots, which suggests lineage-specific evolution of monocots HyPRPs. Based on our previous RNA-Seq study, we selected differentially expressed *OsHyPRPs* genes and used quantitative real-time-PCR (qRT-PCR) to measure their transcriptional responses to biotic (*Magnaporthe* *oryzae*) and abiotic (heat, cold, and salt) stresses and hormone treatment (Abscisic acid; ABA, Methyl-Jasmonate; MeJA, and Salicylic acid; SA) in rice blast susceptible Pusa Basmati-1 (PB1) and blast-resistant near-isogenic line PB1+*Pi9*. The induction of *OsHyPRP16* expression in response to the majority of stresses and hormonal treatments was highly correlated with the number of *cis*-regulatory elements present in its promoter region. *In silico* docking analysis of OsHyPRP16 showed its interaction with sterols of fungal/protozoan origin. The characterization of the *OsHyPRP* gene family enables us to recognize the plausible role of *OsHyPRP16* in stress tolerance.

## 1. Introduction

Being sessile in nature, plants are continuously exposed to biotic and abiotic stresses that adversely affect their productivity. The plant defense response against biotic stresses can be categorized into pathogen-associated molecular patterns (PAMP)-triggered immunity (PTI) and effector-triggered immunity (ETI) [1]. In both PTI and ETI, the activation of a complex network of signaling cascade pathways leads to the induction of resistance response mediated through pathogenesis-related (*PR*) genes, reactive oxygen species (ROS) and secondary metabolites [2]. The initiation of PR proteins against various biotic stresses has been reported in different plant species [3,4]. Among the 17 classes of PR proteins [5], the 8-cysteine motif (8CM) containing PR proteins belong to the prolamin superfamily, which can be further categorized into subfamilies including non-specific lipid transfer proteins (nsLTPs), 2S albumins, alpha-amylase/trypsin inhibitors, and hydrophobic seed proteins [6,7]. The 8CM is usually 90–100 amino acid residues long with a conserved pattern of cysteine residues spaced as C-X_n_-C-X_n_-CC-X_n_-CXC-X_n_-C-X_n_-C [6,8]. The 8CM proteins are different from the other eight cysteine residue-containing proteins, such as lectins and agglutinin, based on the size, the pattern of cysteine spacing, an array of disulfide bonds, and hydrophobicity profile [9].

In several 8 CM containing proteins, the proline-rich repeats precede the 8CM, and such proteins are classified as HyPRPs. However, the N-terminal domain of HyPRPs resembles the structural cell-wall proline-rich proteins (PRPs) [10,11], they lack the characteristic amino acid motifs of PRPs and are highly variable in their size and proline repeats composition [8,12]. The presence of atypical N-terminal proline-rich repeat domain and typical C-terminal 8CM domain categorize the HyPRPs together with the 8CM containing proteins of prolamin superfamily [8]. The structural analysis of a typical 8CM domain-containing protein, nsLTP, revealed that four disulfide bonds between eight cysteine residues are essential for a stable hydrophobic cavity that can accommodate various lipids and hydrophobic ligands with high specificity and plasticity [13,14,15].

The expression profile of *HyPRPs* has been primarily used to infer their functions. For example, the stress-inducible expression of *BnPRP* (*Brassica napus*), *CcHyPRP* (*Cajanus cajan*), *EARLI1* (*Arabidopsis*), *GhHyPRP3* (*Gossypium hirsutum*), *GhHyPRP4*, *JsPRP1* (*Juglans sigillata*), *MfHyPRP* (*Medicago falcata*), *MsPRP2* (*Medicago sativa*) suggest their role in abiotic stress tolerance. [16,17,18,19,20,21,22,23,24]. Similarly, the induced expression of *AtDHyPRP1* (Arabidopsis), *CcHyPRP*, *JsPRP1*, *GmHyPRP* (*Glycine max*) and *SbPRP* (*Glycine max*) in response to bacterial or fungal or viral pathogens indicate their role towards biotic stresses [24,25,26,27,28]. In response to various signaling molecules such as phytohormones, H_2_O_2_, NO, and other oxidative stress-inducing molecules, the altered expression of *HyPRPs* including *JsPRP1*, *MfHyPRP*, *SbPRP*, and *SlHyPRP1* (*Solanum lycopersicum*), suggests their role either directly or indirectly in the defense-related signaling pathways [23,24,26,29].

The functional analysis of *HyPRPs* suggests their diverse roles in biotic and abiotic stress tolerance, morphogenesis, and cellular and developmental processes [9,19,20,30,31,32,33,34]. The functional characterization of *HyPRP* genes, including *EARLI1*, *PtrPRP* (*Poncirus trifoliata*), and *GhHyPRP4*, suggests their positive role in the freezing or cold tolerance [19,21,35,36]. The overexpression of *CcHyPRP*, *GhHyPRP3*, *JsPRP1*, and *MfHyPRP* were found to enhance tolerance toward multiple abiotic stresses, such as salinity and cold stress [20,22,23,37]. In contrast, tomato *SlHyPRP1* and *SpHyPRP1* (*Solanum pennellii*) were found to negatively regulate the stress response against drought, salt, and oxidative stresses in tomato [29]. The Arabidopsis HyPRP, AZI1, was found to play an essential role as a vital component in systemic acquired resistance (SAR), during *Pseudomonas syringae* infection [38]. Similarly, tobacco overexpressing *JsPRP1* exhibited strong resistance against *Colletotrichum gloeosporioides* infection [24]. Conversely, the *CaHyPRP1* (*Capsicum annuum*) and *GbHyPRP1* (*Gossypium barbadense*) were found to negatively regulate the basal defense during biotic stresses, possibly through suppression of ROS [39,40]. All the above studies highlighted the importance of *HyPRPs* in biotic and abiotic responses, and therefore, it is of great significance to identify and characterize the *HyPRPs* in plants. Although Dvořáková et al. [12] identified members of the *HyPRPs* gene family in seven plant species, including rice, they explained the comparative sequence diversity among the *HyPRPs* gene family members.

Rice (*Oryza sativa* L.) is a cereal that is widely consumed as a staple food; therefore, sustainable rice production is a key factor towards insuring food security for our world’s continuously-growing population. It is essential to reduce the losses associated with various biotic and abiotic stresses to achieve sustainability in rice production. Owing to the significance of *HyPRPs* in biotic and abiotic stress tolerance, in the present investigation, we carried out genome-wide identification, comparative phylogeny and duplication analysis on the *HyPRP* gene family in rice. Furthermore, we performed the qRT-PCR based expression profiling of rice *HyPRPs* genes in response to biotic and abiotic stresses, and phytohormone treatment in rice blast susceptible PB1 and near-isogenic blast-resistant rice line PB1+*Pi9* [41,42,43]. The present investigation provides a basic framework that can be explored to unravel the biological significance of rice *HyPRPs* and to target the putative candidate genes of this 8CM gene family to devise strategies for the development of climate-resilient crop plants.

## 2. Results and Discussion

### 2.1. Identification and Annotation of Putative OsHyPRPs in Rice

**Figure 1:**
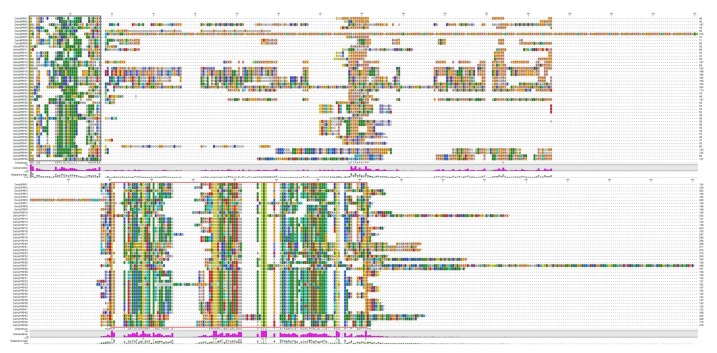
The multiple sequence alignment of OsHyPRPs showing conserved regions. The conserved amino acid residues in the black box are N-terminal signal peptides (may extend further for several proteins due to alignment constrains), while the C-terminal 8CM of OsHyPRPs is marked in the red box. The 8CM region is typical to the other 8CM protein with a conserved eight cysteine skeleton. The region in-between the black and red boxes showed the non-conserved proline-rich region, which is atypical to the proline-rich cell wall structural proteins.

The scanning of hidden Markov model (HMM) profile resulted in the identification of 65, 156 and 134 proteins containing Hydrophob_seed (PF14547), Tryp_alpha_amyl (PF00234), and LTP_2 (PF14368) domains, respectively. The PfamScan was used to confirm the presence of these domains, which resulted in the identification of 50, 130, and 131 proteins with Hydrophob_seed domain, Tryp_alpha_amyl domain, and LTP_2 domain, respectively. By combining the results, a total of 179 proteins were detected as unique proteins, where four proteins (LOC_Os07g11310.1, LOC_Os07g12080.1, LOC_Os07g11630.1, and LOC_Os07g11650.1) based on the BLAST similarity were found to be trypsin-alpha amylase inhibitors or cereal storage proteins and therefore removed from the further analysis. We manually inspected the sequences of the remaining 175 candidate proteins and excluded 126 proteins, which lacked proline-rich repeats and 8CM. Additionally, we removed three proteins without N-terminal signal peptides. Using this stringent process, we identified a total of 46 putative OsHyPRPs encoded by 45 *OsHyPRPs* genes in rice, which showed characteristic N-terminal signal peptides followed by proline residues repeats and C-terminal conserved 8CM motifs. However, in the previous studies by Dvorakova et al. [12] and Boutrot et al. [44], identified 31 and 21 genes, respectively, encoding HyPRPs in the whole rice genome. Such a difference in the number of identified *HyPRPs* may be due to the use of different identification approaches and the continuous refinement of the rice genome. The multiple sequence alignment of OsHyPRP protein sequences showed the conservation of the eight-cysteine skeleton in the 8CM region, while the proline-rich region was found to be highly non-conserved (Figure 1).

The Physico-chemical characteristics of the 46 identified OsHyPRPs are summarized in Table 1. The average molecular mass and the theoretical pI of mature OsHyPRP proteins was estimated to be 16.3 kDa and 7.3 pI, respectively. The signal peptide length for OsHyPRP proteins varied from 20 to 32 amino acid residues. Using signalP, we predict that the majority of OsHyPRPs targets to the secretory pathway, with few exceptions, where OsHyPRP24 and OsHyPRP46 target to the mitochondria, while OsHyPRP43, OsHyPRP44, and OsHyPRP45 target to the chloroplast (Table 1).

### 2.2. Chromosomal Location, Gene Duplication and Structural Analysis of OsHyPRPs

**Figure 2:**
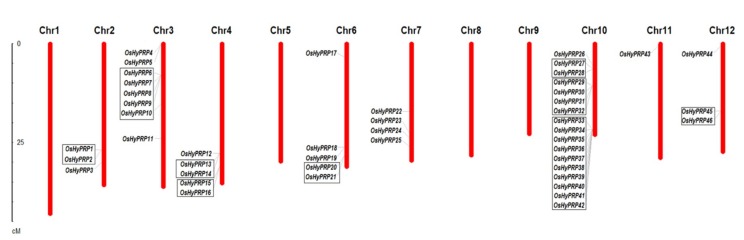
Chromosomal mapping and clustering of 46 *OsHyPRP* gene models in rice chromosomes. The boxes indicate the genes present within the region of 200 Kbp to form a gene cluster.

The mapping of 46 *OsHyPRP* genes on 12 rice linkage groups (LGs) revealed their biased distribution (Figure 2). Out of 46 *OsHyPRP* genes, 35 genes (>75%) are present on LG3, LG4, LG6, and LG10 with 8, 5, 5, and 17 *OsHyPRP* genes, respectively, while the LG1, LG5, LG8, and LG9 are devoid of *OsHyPRPs* (Figure 2). The gene cluster analysis showed that 31 *OsHyPRP* genes (>67%) are present in nine clusters, distributed on six LGs with the largest gene cluster of 10 genes on LG10. The gene duplication analysis of *OsHyPRP* genes revealed the presence of 11.1%, 20%, 8.8%, and 62% of singleton, dispersed, proximal, and tandem duplication, respectively. Of the total 46 *OsHyPRP* genes, 21 are tandemly duplicated (Supplementary Figures S1 and S2), this indicates that ongoing tandem duplications in the rice genome [12,45] might be causing *OsHyPRP* gene family expansion in rice. Results from the gene structure analysis of *OsHyPRP* genes showed that the majority of them (33 genes, 71%) are intron-less, while the remaining genes (13 genes, 28%) have introns that vary from 1 to 3 in number (Supplementary Figure S3).

### 2.3. Phylogenetic Analysis of the OsHyPRP Family among Different Plant Species

**Figure 3:**
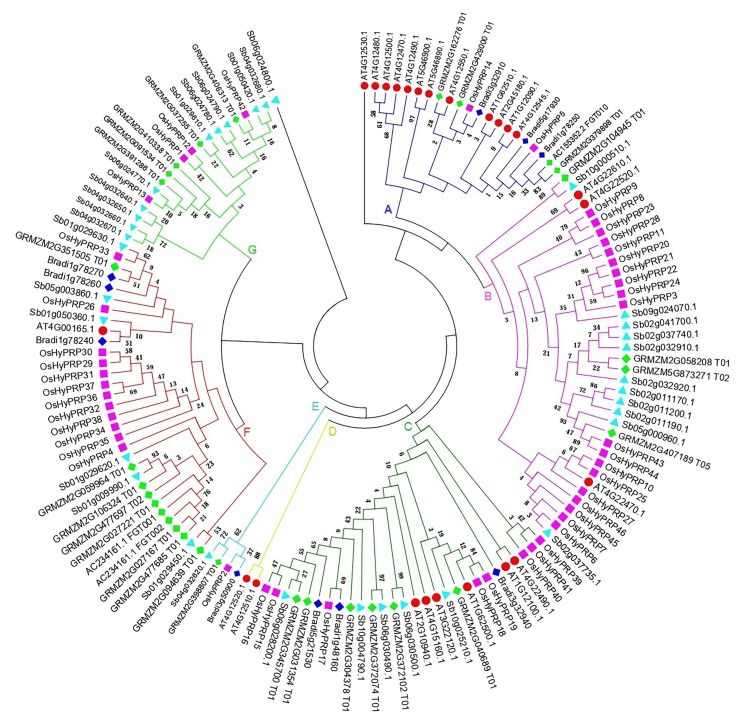
Phylogenetic relationship of rice HyPRPs with that of Arabidopsis, maize, sorghum and *B. distachyon.* The 8CM region of all the HyPRPs was used to construct the phylogenetic tree. The multiple sequence alignment and phylogenetic tree construction were performed with MEGA6.06 using maximum likelihood method with 500 bootstrap replicates. The different shapes with color code represent the HyPRPs of different plant species; red sphere for Arabidopsis, magenta square for rice, cyan triangle for sorghum, lawn-green diamond for maize, and blue diamond for *Brachypodium*. The one protein each from sorghum and Arabidopsis does not group with other HyPRPs and thus considered as highly divergent outliers.

To analyze the phylogenetic relationships of the rice OsHyPRPs with that of HyPRPs of other plant species, a total of 144 HyPRP proteins; 46 of rice, 24 of Arabidopsis, 30 of maize, 34 of sorghum, and 10 of *Brachypodium distachyon* were used to construct a maximum-likelihood phylogenetic tree with 500 bootstrap replicates. The phylogenetic tree categorized all the 144 HyPRP proteins into seven distinct clades (A to G; Figure 3). Notably, the majority of OsHyPRPs were found to cluster together in separate sub-clades along with the HyPRPs of maize and sorghum in clade B and F (19 and 12 OsHyPRPs, respectively), while the Arabidopsis HyPRPs were mainly present in clade A and C (Figure 3). The clustering of the HyPRPs of rice, maize, and sorghum together, and separation from Arabidopsis HyPRPs may indicate their early divergence during monocotyledon lineage-specific evolution. The random distributions of *B. distachyon* HyPRPs in several clades offers an exception to this hypothesis, which may be due to high sequence diversity and low numbers of HyPRPs in *B. distachyon*. The phylogenetic analysis also revealed that the majority of OsHyPRPs clustered together into sub-clades are also tandemly duplicated and found to present as gene clusters on rice LGs (Figure 2 and Figure 3, and Supplementary Figure S2).

### 2.4. OsHyPRPs Expression Profile in Response to M. oryzae

**Figure 4:**
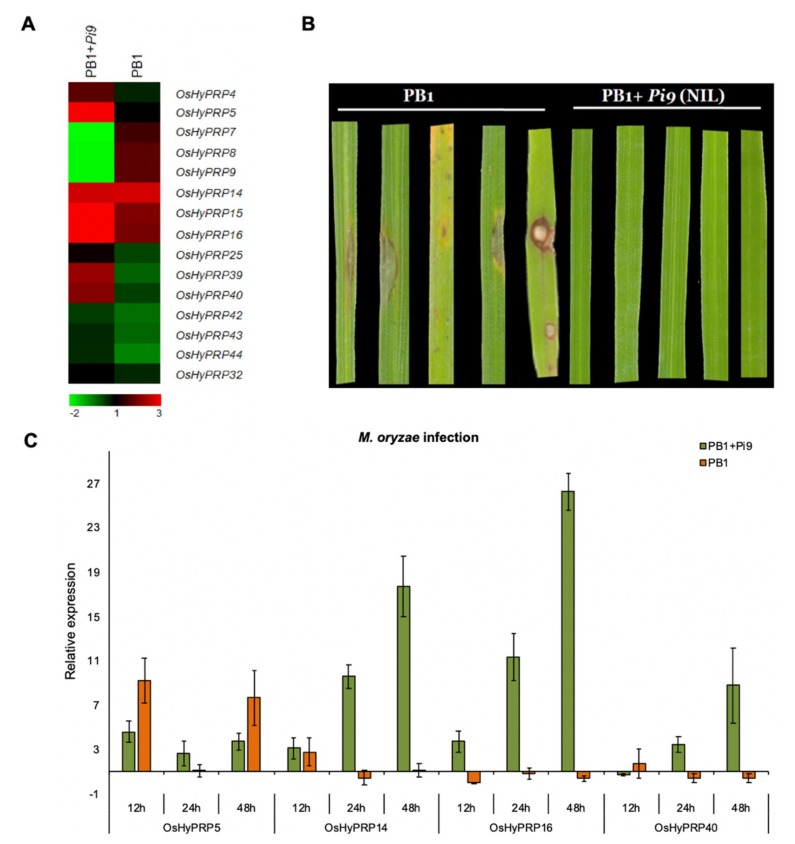
The expression of *OsHyPPR* genes in response to *M. oryzae*. (**A**) The heat map for the expression profile of *OsHyPRP* genes as determined through RNA-Seq analysis of PB1 and PB1+*Pi9* in response to *M. oryzae* after 24 hpi. The expression shown here is the log2 fold change value as compared to mock-treated PB1 and PB1+*Pi9*. (**B**) The picture is showing disease lesions in PB1 after seven days of *M. oryzae* inoculation, while no disease lesions were present in PB1+*Pi9* (**C**) The qRT-PCR based expression profile of four *OsHyPRP* genes showed up-regulated expression in RNA-Seq analysis. The calculated expression level was relative to the untreated respective rice lines. The experiment was performed with three technical replicates of three independent biological replicates. The error bars represent the standard error of the means of three independent biological replicates. The rice *elongation factor-1A* (*OsEF-1a*) gene was used to normalize the qRT-PCR expression data.

Under natural environmental conditions, plants are vulnerable to various biotic and abiotic stresses that severely affect their productivity. Sometimes, the exposure to abiotic stresses compromises the plant’s resistance toward biotic stress through reorientation of plant-pest interaction [46,47]. Moreover, the biotic agent directly uses the plant resources, which eventually leads to reduced plant vigor [48]. Therefore, it is of great significance to identify the key regulators that drive the defense response of plants towards biotic and abiotic stresses. The rice blast disease is a major risk to sustainable rice production since it causes 10–30% of global loss annually [49]. In one of the previous studies from our group, we performed the RNA-Seq based transcriptome analysis for *M. oryzae* inoculated rice blast susceptible PB1 and blast-resistant PB1+*Pi9* rice lines at 24 h post-infection (hpi) [43]. This data is available at the Gene Expression Omnibus (GEO) dataset with accession number GSE81906 (www.ncbi.nlm.nih.gov/gds/?term=GSE81906[Accession]). Using this data, we analyzed the expression of *OsHyPRPs*, where out of 46 *OsHyPRPs* genes 15 were found to exhibit expression in at least one of the samples, while the rest of the 31 genes showed no expression. A higher number of *OsHyPRP* genes were found to have up-regulated expression in PB1+*Pi9* when compared to PB1, after 24 hpi with *M. oryzae* (Figure 4A). Based on the transcriptome analysis, we selected six highly-expressed *OsHyPRP* genes (*OsHyPRP5*, *OsHyPRP14*, *OsHyPRP15*, *OsHyPRP16*, *OsHyPRP39*, and *OsHyPRP40*) for their qRT-PCR based expression profiling in response to biotic & abiotic stresses, and hormone treatment. Out of the six selected *OsHyPRP* genes, we observed the qRT-PCR based expression of four *OsHyPRP* (*OsHyPRP5*, *OsHyPRP14*, *OsHyPRP16*, and *OsHyPRP40*) genes.

The qRT-PCR analysis of collected samples was performed only after the appearance of disease lesions at seven days post-inoculation of *M. oryzae* in PB1 (Figure 4B). The expression of all the four *OsHyPRPs* showed induction in response to *M. oryzae* in PB1+*Pi9* (Figure 4C). However, the expression of *OsHyPRP5* was early inducible, a similar expression pattern was observed in both PB1+*Pi9* and PB1 upon *M. oryzae* inoculation (Figure 4C). In addition to *OsHyPRP5*, *OsHyPRP14* and *OsHyPRP16* also exhibited early induced expression (>three-fold up-regulation) at 12 hpi in response to *M. oryzae* inoculation (Figure 4C). Comparatively, among the other genes, the expression of *OsHyPRP16* was strongly induced upon *M. oryzae* inoculation in PB1+*Pi9*. In the blast-susceptible PB1 rice line, the expression of *OsHyPRP14*, *OsHyPRP16*, and *OsHyPRP40* either remained at the basal level or down-regulated in response to *M. oryzae*, except for 2.8-fold up-regulation of *OsHyPRP14* at 12 hpi (Figure 4C). This finding shows that the presence of *Pi9* positively regulates the expression of *OsHyPRPs*, and this induced expression may be involved in the enhanced defense response in rice blast resistant PB1+*Pi9* plants. Additionally, our qRT-PCR based expression patterns of *OsHyPRPs* are comparable with that of the RNA-Seq transcriptome data of Jain et al. [43] (Figure 4A,C) and thus further corroborate the up-regulation of *OsHyPRP* genes in PB1+*Pi9* as compared to PB1, upon *M. oryzae* inoculation.

Similar to the present study, the early induced up-regulation of Arabidopsis *AtDHyPRP1* has been observed in response to *P. syringae* [28]. Likewise, the *C. gloeosporioides* infected walnut was found to show early induced expression of the *JsPRP1* gene [24]. In soybean, the expression of *GmHyPRPs* and *SbPRP* was found to be up-regulated in response to *Phakopsora pachyrhizi* [27] and soybean mosaic virus [26], respectively. Several studies have reported the role of *HyPRPs* to confer the disease resistance towards different plant pathogens. Recently, Liu et al. [24] found that the overexpression of the walnut *HyPRP* gene, *JsPRP*, in tobacco enhances the resistance against *C. gloeosporioides* in transgenic plants. Additionally, using *in vitro* assay, the recombinant JsPRP protein was found to show antifungal activity towards different fungal pathogens such as *C. gloeosporioides, Gibberella moniliformis*, *Botryosphaeria dothidea*, and *Fusarium solani* [24]. The similar antifungal activity of Arabidopsis HyPRP protein, EARLI1, against *Saccharomyces cerevisiae* has been reported by Li et al. [50]. Additionally, the exogenous application of recombinant EARLI1 was found to inhibit the conidial germination and hyphae growth of *Botrytis cinerea* and *Fusarium oxysporum* [50]. In a recent study, the *CcHyPRP* overexpressing transgenic rice lines exhibit enhanced tolerance towards both biotic and abiotic stresses, where enhanced tolerance towards biotic stresses was suggested to be plausibly contributed by the higher endochitinase activity in transgenic rice plants [37]. Jung et al. [38] illustrated the role of EARLI-type HyPRP, AZI1, as a component of plant systemic immunity, where AZI1 may act to regulate and/or directly translocate the mobile signals during SAR in response to biotic stress. All these findings suggest that the *HyPRPs* perform a diverse role in enhancing plant tolerance towards different biotic stresses.

In addition, a few other studies have also reported negative effects of *HyPRPs* on plant’s resistance against biotic stress. In one recent study, using a gain- and loss-of-function approach, Yang et al. [40] suggested the role of *GbHyPRP1* as a negative regulator of disease resistance in cotton against pathogenic fungi *Verticillium dahlia*. Using a similar approach, Yoem et al., [39] characterized two *HyPRP* genes; *CaHyPRP1* from *C. annuum* (pepper) and *NbHyPRP1* from *Nicotiana benthamiana*. The transient overexpression of *CaHyPRP1* in *N. benthamiana* enhances the disease susceptibility towards virulent *P. syringae* pv. *tabaci*. In contrast, the silencing of *CaHyPRP1* and *NbHyPRP1* resulted in the enhanced basal defense against virulent and avirulent plant pathogens through suppression of pathogen-induced cell death [39]. Based on these results, Yoem et al. [39] suggested that *CaHyPRP1* and *NbHyPRP1* perform the dual function as a positive regulator of cell death and negative regulator of plant basal defense against pathogens. Together, in the light of previous findings, the early and strong induction of *OsHyPRPs*, particularly *OsHyPRP14* and *OsHyPRP16* genes in PB1+*Pi9* suggests that these genes may contribute towards the enhanced tolerance against *M. oryzae* in the blast-resistance PB1+*Pi9* rice line.

### 2.5. OsHyPRPs Expression Profile under Abiotic Stress

**Figure 5:**
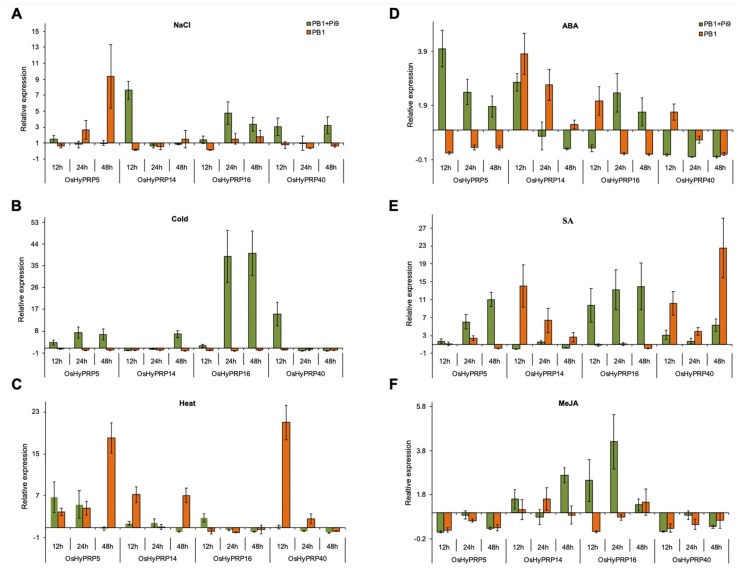
The qRT-PCR based expression of four selected *OsHyPPR* genes in PB1 and PB1+*Pi9*, in response to stress such as NaCl (**A**), cold (**B**), and heat (**C**), and hormone treatments including ABA (**D**), SA (**E**) and MeJA (**F**). The calculated expression level was relative to the untreated rice seedlings of respective rice lines. The experiment was performed with three technical replicates of three independent biological replicates. The error bars represent the standard error of the means of three independent biological replicates. The rice *elongation factor-1A* (*OsEF-1a*) gene was used to normalize the qRT-PCR expression data.

Abiotic factors such as salt, drought, and temperature are the key environmental cues which directly affect the plant’s geographical distribution, physiology, and productivity [47,48,51,52,53,54,55,56]. In the present investigation, the expression profiling of *OsHyPRP* genes under salt, cold, and heat stress revealed their differential expression. In PB1+*Pi9*, under salt stress, all the genes exhibit induced expression, except for *OsHyPRP5* that remains at the basal level at all the time points (Figure 5A). Among the early induced *OsHyPRPs*, the *OsHyPRP14* exhibit more than seven-fold up-regulation at 12 h of salt treatment, which reduced to the basal level at subsequent time points (Figure 5A). Although the *OsHyPRP16* shows no early induction, its expression was more than three-fold up-regulated at 24 h and 48 h of salt treatment in PB1+*Pi9* (Figure 5A). In the case of PB1, only the *OsHyPRP5* exhibit increased expression in response to salt stress, while the other genes remain either at the basal level or down-regulated as compared to untreated control (Figure 5A).

Surprisingly, we found that all four *OsHyPRP* genes were down-regulated in PB1 under cold stress. In contrast to PB1, the *OsHyPRP5* and *OsHyPRP16* exhibit early induced and consistently up-regulated expression in PB1+*Pi9* under cold stress, whereas *OsHyPRP16* shows comparatively strong up-regulation (>39-fold at 24 h and 48 h) (Figure 5B). Although the *OsHyPRP14* and *OsHyPRP40* were also found to be cold-inducible, their expression shows up-regulation only at 48 h (6.8-fold) and 12 h (15.1-fold), respectively (Figure 5B). In contrast to cold stress, the majority of *OsHyPRPs* exhibits up-regulation in PB1 under heat stress, except for *OsHyPRP16*, whose expression remains constantly down-regulated (Figure 5C). In PB1, the expression of *OsHyPRP5* showed a continuous increase (four-fold at 12 h to 18-fold at 48 h) in response to heat stress, while in PB1+*Pi9* it showed a reciprocal pattern of expression (6.7-fold at 12 h to 0.7-fold at 48 h) (Figure 5C). Besides *OsHyPRP5*, the *OsHyPRP14* (>seven-fold at 12 h and 48 h) and *OsHyPRP40* (21-fold at 12 h) also exhibit up-regulation in PB1 under heat stress, while in contrast, their expression was remained either at the basal level or down-regulated in PB1+*Pi9* (Figure 5C). In PB1+*Pi9*, the *OsHyPRP16* was the only gene that showed differentially up-regulated expression (2.8-fold) at 12 h of heat stress, as compared to PB1 (Figure 5C).

In summary, the expression of *OsHyPRP14*, *OsHyPRP16*, and *OsHyPRP40* showed differential up-regulation in PB1+*Pi9*, as compared to PB1, during all the abiotic stresses (Figure 5A–C). Similar to the present study, the expression of pigeonpea *CcHyPRP* showed strong induction in the leaf and root tissues in response to different abiotic stresses such as drought, salt, heat, and cold [20,57]. In cotton, the strongly up-regulated expression of *GhHyPRP4* in cold stress, and *GhHyPRP3* in several abiotic stresses such as dehydration, cold and salt stress has been observed [21,22]. Likewise, the cold-inducible expression of Arabidopsis *HyPRPs*, namely *EARLI1* and *AZI1*, has been reported [58,59,60], where the *AZI1* was also observed to show salt stress-inducible expression [61]. Similarly, in response to cold and salt stress, the *P. trifoliata PtrPRP* exhibits induced expression [36]. The expression of *msa CIC* (*HyPRP* gene) in cold-tolerant *M. sativa* [16] and, *MfHyPRP* in *M. falcata* was found to be cold-inducible [23]. Furthermore, the *MfHyPRP* was found to show an inducible expression in response to drought stress and, hydrogen peroxide and nitric oxide treatment; however, its expression remains low under salt stress [23]. Similarly, the soybean *SbHyPRP* was found to show inducible expression only under low salt conditions, while its expression remained low under high salt conditions [26].

Several functional studies have been performed in the last two decades to characterize the role of *HyPRPs* in response to abiotic stresses. The EARLI-type *HyPRPs* in Arabidopsis were found to play a significant role in cold and salt stress tolerance [19,59,60]. The RNA interference (RNAi) based suppression of *EARLI* resulted in the reduced freezing tolerance in *EARLI* suppressed Arabidopsis lines [19]. Moreover, higher electrolyte leakage in the *EARLI1* knockdown Arabidopsis, as compared to *EARLI1* overexpressing Arabidopsis, suggests that *EARLI1* enhances the freezing tolerance through reduction of freezing-induced cellular damage [35]. Additionally, the overexpression of *EARLI1* in Arabidopsis plants conferred enhanced tolerance towards salt stress [59]. Similar to *EARLI1*, its homolog in Arabidopsis, *AZI1*, was also found to enhance the salt and freezing tolerance in *AZI1* overexpressing Arabidopsis [60,61]. The AZI1 was found to act as a direct target of mitogen-activated protein kinase MPK3, where the presence of MPK3 is essential for strong and robust tolerance towards salt stress [61]. The overexpression of *MfHyPRP* in tobacco conferred enhanced tolerance towards freezing and chilling stress, in addition to the methyl viologen induced oxidative stress [23]. Similarly, the ectopic expression of *CcHyPRP* in yeast, Arabidopsis, and rice resulted in the enhanced tolerance towards multiple abiotic stresses, including drought, salt, and heat stresses [20,37]. Moreover, under abiotic stress conditions, the transgenic rice overexpressing *CcHyPRP* showed a higher survival rate with more productivity when compared to wild type [37]. Using the RNAi approach, Peng et al. [36] showed that the *PtrPRP* suppressed *P. trifoliate* plants were more susceptible to cold stress than wild type plants, which indicates the role of *PtrPRP* as a positive regulator of cold tolerance. In contrast, Li et al. [29] demonstrated the negative role of tomato *SpHyPRP1* on abiotic stresses tolerance, where *SpHyPRP1* was found to be responsible for the reduced ROS-scavenging and thus compromised abiotic stress tolerance, which is believed to be contributed by ROS-scavenging [62]. All the above previous findings suggest that *HyPRPs* are involved either as a positive regulator [20,37,59,60] or negative regulator [29,39] of plants stress tolerance. In the present study, the strongly induced expression of *OsHyPRP16* and *OsHyPRP40* was found to be more consistent at least during cold and salt stresses, which indicates their possible role in defense response towards these abiotic stresses.

### 2.6. Expression Profiling of OsHyPRPs under Phytohormone Treatment

Phytohormones are small signaling molecules that perform diverse functions such as regulation of cellular and developmental processes during the plant lifespan [63,64,65]. The accumulation of phytohormones such as ABA, SA, and JA (Jasmonic acid), and their cross-talk accompanied the stress tolerance in plants [66,67,68,69,70]. Since the hormones signaling pathways are crucial for biotic and abiotic stress responses, we performed the expression profiling for selected *OsHyPRP* genes in response to stress-related hormones namely, ABA, SA, and MeJA.

In ABA-treated PB1+*Pi9*, the *OsHyPRP5* (four-fold) and *OsHyPRP14* (>2.7-fold) exhibit early induced up-regulated expression at 12 h (Figure 5D). The expression of *OsHyPRP16* was found to show up-regulation (2.4-fold) only at 24 h in PB1+*Pi9.* Notably the expression of *OsHyPRP14* and *OsHyPRP16* follow the similar temporal expression pattern in PB1+*Pi9* under salt stress and ABA treatments (Figure 5A,D). Unlike others, the expression of *OsHyPRP40* constantly remained down-regulated in PB1+*Pi9* (Figure 5D). In the case of ABA-treated PB1, the expression of *OsHyPRP5* constantly remained down-regulated (Figure 5D). The *OsHyPRP14* follows a similar decreasing expression pattern in both PB1+*Pi9* and PB1, with a comparatively abrupt decrease in the case of PB1+*Pi9* (Figure 5D). The expression of *OsHyPRP16* and *OsHyPRP40* was found to show up-regulation only at 12 h of ABA treatment in PB1 (Figure 5D). In summary, the ABA treatment resulted in the differential and induced expression of *OsHyPRP5* and *OsHyPRP16* in PB1+*Pi9*, as compared to PB1. Similar to our study, the ABA treatment resulted in a strong and early induced expression of *CcHyPRP1* in pigeonpea roots, *MfHyPRP* in *M. falcate*, and *PtrPRP* in *P. trifoliata* [20,23,36]. In contrast, the *BnPRP* in *B. napus*, *GhHyPRP4* in cotton and *SbPRP* in soybean, and *SpHyPRP1* in tomato were found to be down-regulated upon ABA treatment [18,21,26,29]. Since the suppression of negative regulators or overexpression of positive regulators of ABA response is known to confer drought tolerance [19,71], the down-regulation of *SpHyPRP1* in response to exogenous ABA, and the reduced growth along with enhanced salt and drought sensitivity in *SpHyPRP1* overexpressing transgenic tomato suggest the negative role of *SpHyPRP1* in ABA signaling [29]. In the present analysis, the *OsHyPRP5* and *OsHyPRP16* were differentially induced in ABA-treated PB1+*Pi9* as compared to PB1 (Figure 5D), which indicates the positive effect of *Pi9* on their expression. However, it is required to validate the role of *OsHyPRP5* and *OsHyPRP16* in ABA-mediated salt and drought stress response.

In response to SA treatment, the *OsHyPRP5* and *OsHyPRP16* exhibit up-regulated expression in PB1+*Pi9*, while their expression remains either at the basal level or down-regulated in PB1, except for the 2.4-fold up-regulation of *OsHyPRP5* at 24 h (Figure 5E). Although the *OsHyPRP40* also exhibit up-regulated expression in response to SA, it shows a similar expression pattern in both PB1+*Pi9* and PB1, with comparatively strong up-regulation in the latter (Figure 5E). Unlike other genes, the expression of *OHyPRP14* was only up-regulated in PB1 upon SA treatment, while it remained either at the basal level or down-regulated in PB1+*Pi9* (Figure 5E). Similar to ABA treatment, the expression of *OsHyPRP5* and *OsHyPRP16* was also induced in SA-treated PB1+*Pi9* (Figure 5D,E). In MeJA-treated PB1+*Pi9*, only the *OsHyPRP16* exhibit early and strong induction (2.5-fold at 12 h) with peak expression at 24 h (4.2-fold) (Figure 5F). However, the expression of *OsHyPRP14* also showed up-regulation (2.7-fold at 48 h) in the later stages of MeJA treatment in PB1+*Pi9* (Figure 5F). Meanwhile, in PB1, the expression of *OsHyPRP14* and *OsHyPRP16* remains either at the basal level or down-regulated in response to MeJA (Figure 5F). Unlike other hormone treatments, the expression of *OsHyPRP5* and *OsHyPRP40* remains low in both MeJA-treated rice lines (Figure 5F). Altogether, in response to each ABA, SA, and MeJA treatment in PB1+*Pi9*, the *OsHyPRP16* is the only gene that showed up-regulated expression, at least in one of the time points of hormone treatment.

Only a few previous studies are available that have analyzed the expression profile of *HyPRPs* in response to SA and MeJA/JA treatments. In soybean, the expression of *SbPRP* was found to show rapid induction upon SA treatment, while in contrast, its expression shows down-regulation in response to MeJA treatment [26]. In the present investigation, the *OsHyPRP5* and *OsHyPRP40* showed a similar expression behavior in response to SA and MeJA treatment in PB1+*Pi9* (Figure 5E, F). Li et al., [28] reported the induced expression of *AtDHyPRP1* in response to MeJA and SA treatment as well as in response to *P. syringae*. We also observed the induced expression of *OsHyPRP16* under biotic stress and hormone treatments, including ABA, SA, and MeJA (Figure 5D–F). In a recent study, the expression of cotton *GbHyPRP* was strongly down-regulated in response to SA treatment, while it was significantly up-regulated in each ABA, JA, and ethylene treatment [40]. Based on the similarity in the expression profile of *GbHyPRP* in response to SA and *V. dahlia* inoculation, Yang et al. [40] proposed a negative role of *GbHyPRP* towards *V. dahlia* resistance in cotton via a SA-mediated signaling pathway [40]. Intriguingly, in the present study, the up-regulation of *OsHyPRP16* also follows a similar expression pattern in SA-treated and *M. oryzae* inoculated PB1+*Pi9* (Figure 4 and Figure 5E) [41,42]. Based on this expression similarity, we also suspect that *OsHyPRP16* and SA-mediated signaling might be involved to confer blast-resistance in PB1+*Pi9*, where *Pi9* is supposed to have a central role in activating the basal defense pathway.

### 2.7. Analysis of Cis-Regulatory Elements in Four OsHyPRP Promoter Sequences

**Figure 6:**
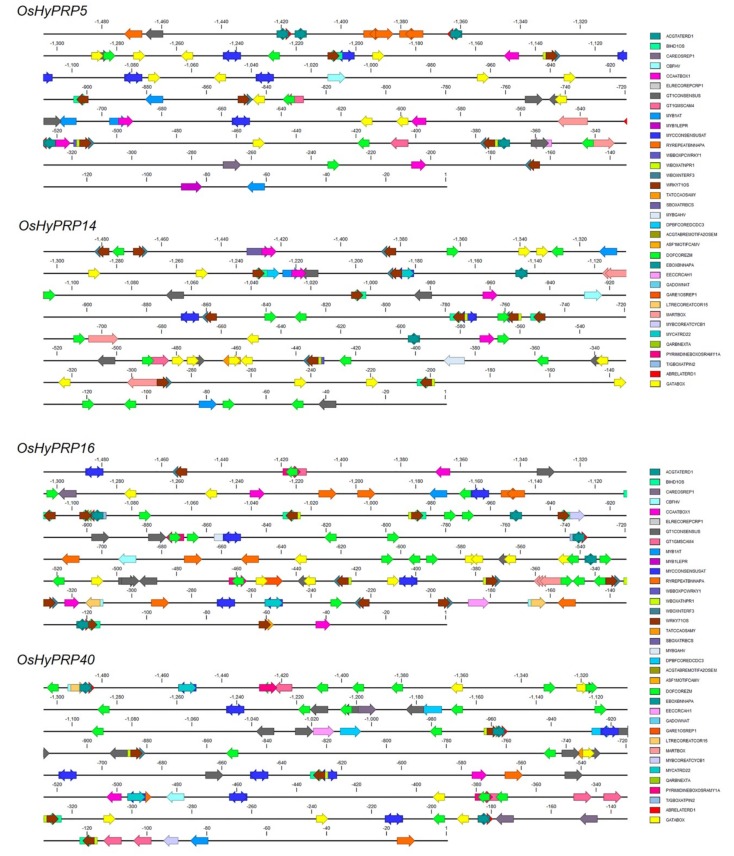
In silico analysis of the promoter region of four *OsHyPRPs* shows the presence of different *cis*-regulatory elements. The 1500 bp region up-stream of ATG start codon was analyzed using the PLACE database webserver. The base immediate upstream of ATG is considered as −1 and the positions of various *cis*-regulatory elements are relative to −1 position. Arrows at the *cis*-regulatory elements indicate the position of elements on either + strand (forward direction) or − strand (reverse direction).

The *cis*-elements act as molecular switches that participate in the transcriptional regulation of genes during different environmental cues [72]. The 1.5 kb upstream DNA sequences of the four selected *OsHyPRP* genes were retrieved and analyzed using the PLACE database. The list of various putative *cis*-regulatory elements present in the upstream sequences is given in Supplementary Table S1. The promoter sequences were found to contain sequence motifs that are responsive to biotic stresses (pathogen, elicitor, and disease resistance responsive elements), abiotic stress (cold, heat, salt, drought, and wounding responsive elements) and hormones (ABA, SA, JA, and GA responsive elements) (Figure 6 and Supplementary Table S1). The detailed analysis of sequence motifs revealed that the promoter sequence of *OsHyPRP16* and *OsHyPRP40* contains the highest number of sequence motifs that are responsive to biotic and abiotic stresses and, hormones treatment. However, the expression of *OsHyPRP40* under different experimental conditions did not show correlation with the number of *cis*-regulatory elements in its promoter sequence, except for *M. oryzae* inoculation and SA treatment (Figure 4C and Figure 5E and Supplementary Table S1). Similarly, in one of the previous studies, besides the absence of cold-responsive elements, the *GhHyPRP4* promoter was found to be cold-inducible as shown by the high activity of *GhHyPRP4* promoter-driven *β-glucuronidase* (GUS) gene in transgenic Arabidopsis under cold stress [21]. In contrast to *OsHyPRP40*, the *OsHyPRP16* exhibits a good correlation between its inducible expression in response to *M. oryzae*, salt, cold, ABA, SA, and MeJA, and the occurrence of different stresses-and hormone-responsive *cis*-regulatory elements in the promoter sequence (Figure 4C and Figure 5 and Supplementary Table S1). In particular, the promoter sequence of *OsHyPRP16* contains the highest number of *cis*-regulatory elements (28 in number) that are involved in pathogen-, elicitors-, and disease resistance-response. The early and strong inducible expression of *OsHyPRP16* in PB1+*Pi9*, after *M. oryzae* inoculation (Figure 4C) and abiotic stresses (Figure 5A–C), and hormone treatment (Figure 5D–F) indicates that this gene may be actively involved in the defense-related pathways.

### 2.8. Protein Structure Prediction and Docking Analysis

The proline-rich N-terminal domain of *HyPRPs* suggests their role as cell-wall structural proteins; however, the expression of *HyPRPs* in different plant tissues implies their significant roles in growth and development [9]. In *HyPRPs*, the presence of atypical N-terminal proline-rich domain and typical 8CM protein C-terminal domain indicate their novel functions that may be more related to 8CM proteins.

**Figure 7:**
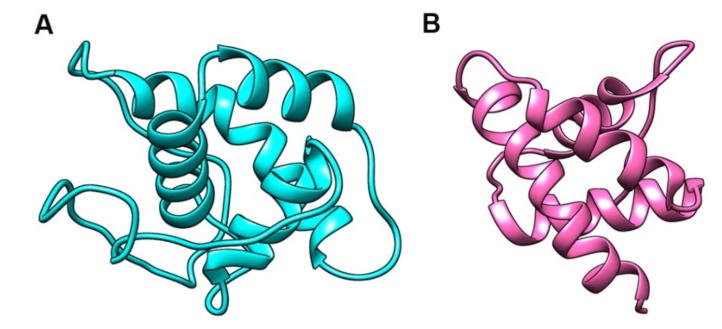
The predicted three-dimensional (3D)-structure of OsHyPRP16 protein. (**A**) Mature OsHyPRP16 (without signal peptide). (**B**) Proline-rich region fragment deleted OsHyPRP16.

The 8CM containing proteins, in particular, nsLTPs are known to bind hydrophobic ligands (sterols and lipids) to perform their biological functions [13,14,15,73]. Owing to the potential role of *OsHyPRP16* in the majority of stresses, it was worthwhile to analyze the binding of OsHyPRP16 protein with different putative ligands. Previously, Yoem et al. [39] illustrated with the help of domain deletion analysis that the 8CM is essential for the cell death-inducing activity of CcHyPRP1. Therefore, in the present investigation, we performed *in silico* docking to analyze the binding of mature OsHyPRP16 (without signal peptide), and fragment deleted OsHyPRP16 (only 8CM) with that of various lipids, lipid-derivatives, fungal cell-wall components, and SA. The deduced three-dimensional (3D) structures of mature and fragment deleted OsHyPRP16 are illustrated in Figure 7. The Ramachandran plot analysis for both the predicted protein structures showed more than 90% of amino acid residues within the energetically allowed region. 

The docking results for the mature OsHyPRP16 showed strong binding with all the putative ligands, while the fragment deleted OsHyPRP16 was found to exhibit either weak or no binding with the putative ligands (Table 2). These results indicate that the presence of the proline-rich region is essential for the strong binding of mature OsHyPRP16 with the putative ligands. The strong putative binding of mature OsHyPRP16 with fungisterol, ergosterol, N-Acetyl glucosamine (NAG), and N-acetyl muramic acid (NAM) may be involved in the PTI (Table 2), where fungal specific sterols may act as PAMPs to trigger the immune response upon fungal attack. Intriguingly, both the mature as well as fragment deleted OsHyPRP16 were found to show strong binding with JA (Table 2). Previously, the tobacco LTP1, an 8CM containing protein, was found to show binding with JA to form LTP1-JA complex, where the exogenous application of LTP-JA complex confers resistance against *Phytophthora parasitica* in tobacco plants [73]. In the present study, the predicted binding of OsHyPRP16 with JA and SA, and the induced expression of *OsHyPRP16* during SA and methyl-ester of JA (MeJA) treatment (Table 2 and Figure 5E,F) suggests a plausible role of OsHyPRP16 to enhance the resistance against *M. oryzae* in rice through SA- and JA-mediated signaling.

## 3. Materials and Methods

### 3.1. Identification and Sequence Analysis of OsHyPRPs

The complete set of rice protein sequences were retrieved from the Rice Genome Annotation Project (RGAP) database available at http://rice.plantbiology.msu.edu. All the retrieved rice protein sequences were searched for the HMM profile of Hydrophobic_seed (PF14547), Tryp_alpha_amyl (PF00234), and LTP_2 (PF14368) using “hmmsearch” program of HMMER v 3.1 (http://hmmer.org), with e value 1e−4. The proteins containing any of the HMM profiles were further scanned using PfamScan program (www.ebi.ac.uk/Tools/pfa/pfamscan/), with parameter; -e_seq 1e-04 -e_dom 1e-04 -clan_overlap, to confirm the presence of domains identified through “hmmsearch” program. All the identified putative rice candidate genes were then aligned and manually analyzed for the presence of 8CMs; C-X_n_-C-X_n_-CC-X_n_-CXC-X_n_-C-X_n_-C, where C stands for cysteine residue and X stands for any other amino acid residue, while n represents the number of amino acid residues. The proteins lacking the essential 8CM and N-terminal signal peptide (predicted through SignalP-5.0; www.cbs.dtu.dk/services/SignalP/, and TargetP1.1; www.cbs.dtu.dk/services/TargetP/) were identified and then excluded from the further analysis. Furthermore, the proteins containing glycosylphosphatidylinositol (GPI) anchored signals (predicted by big-Pi plant server; mendel.imp.ac.at/gpi/plant_server.html) were also excluded from the analysis. The remaining proteins were then manually inspected for the presence of proline-rich regions, as characterized by presence of tandem repeats of (XP)_n_ and/or (XPY)_n_ at least for twice [74]. Finally, the shortlisted proteins containing proline-rich regions in between the N-terminal signal peptide and C-terminal 8CM were named as OsHyPRPs. The pI and molecular weight of identified OsHyPRPs was predicted through the Compute pI/Mw tool (web.expasy.org/compute_pi/).

### 3.2. Chromosomal Mapping and Gene Duplication Analysis of OsHyPRP Genes

The genomic coordinates of the *OsHyPRP* genes were extracted from the general feature format (gff) file (retrieved from https://rapdb.dna.affrc.go.jp/) and were used to schematically represent the intron-exon structure of *OsHyPRP* genes using Gene Structure Display Server (GSDS; gsds.cbi.pku.edu.cn/). The extracted genomic coordinates were also used to map the location of *OsHyPRP* genes on all the 12 rice chromosomes using the MapInspect program (www.plantbreeding.wur.nl/uk/software_mapinspect.html). All the putative genes encoding OsHyPRP proteins were localized on rice chromosomes from 5′ to 3′ based on their genomic coordinates and numbered accordingly from top to bottom on chromosomes 1 to 12 along with prefix *OsHyPRP*. To analyze the duplication events in *OsHyPRP* gene family, all-versus-all BLASTP was performed for 66,338 rice protein sequences with parameter: -evalue 1e-10, -outfmt 6, -num_threads 10 and -max_target_seqs 5. The BLASTP results were then used as input for MCScanX [75] to identify the collinear gene pairs of *OsHyPRP* gene family members in rice with default parameters.

### 3.3. Sequence Alignment and Phylogenetic Analysis

The multiple sequence alignment of full length OsHyPRPs were performed using CLC Genomic Workbench 12.0 (www.qiagenbioinformatics.com/). The 8CM containing C-terminal region of HyPRP proteins of five different plants including rice, Arabidopsis, maize, sorghum, and *B. distachyon* was extracted and used to find the evolutionary relationship among HyPRP proteins of different plant species. The multiple sequence alignment of deduced sequences was generated using MUSCLE [76], with default parameters, and then used for constructing the phylogenetic tree by employing MEGA software version 6.06 using maximum likelihood method based on the JTT model with 500 bootstrap replicates [77].

### 3.4. Biotic and Abiotic Stress and Hormone Treatment

The rice blast contrasting rice lines, namely, PB1 (blast-susceptible) and PB1+*Pi9* (blast-resistant), were used to analyze the expression profile of *OsHyPRP* genes under different stresses and treatments. The seeds of both the rice lines were surface sterilized and germinated on moist filter paper for 4–5 days at 28 °C. The germinated seeds were transferred to the hydroponic system containing half-strength Murashige and Skoog (MS) medium. For abiotic stress and hormone treatment, two-week-old rice seedlings were treated, separately with salt (100 µM, NaCl), ABA (100 µM), MeJA (100 µM), and SA (100 µM) by replacing the hydroponic medium with fresh medium containing respective chemicals. Heat and cold stress were given by incubating the two-week-old rice seedlings at 37 °C and 4 °C, respectively. The leaf samples of treated seedlings were harvested at 0 h, 12 h, 24 h and 48 h intervals after treatment. For biotic stress treatment, we used the method as followed by Jain et al. [43]. Briefly, the 22 days old rice plants were sprayed with *M. oryzae* (Mo-nwi-53) spore suspension (10^5^ spores/mL) containing 0.25% gelatin. Leaf tissues of infected rice plants were collected at 12 hpi, 24 hpi, and 48hpi. Some *M. oryzae* infected plants of each rice line were kept under observation and recorded for disease progression after seven days post-inoculation, using 0–5 disease rating scale [78]. All the treatments were performed in three biological replicates, and the collected tissue samples were immediately frozen in liquid nitrogen and stored at −80 °C till further use.

### 3.5. RNA Extraction and Real-Time Quantitative Reverse Transcription PCR

Total RNA from harvested samples were isolated using Sigma’s Spectrum Plant Total RNA Kit (Sigma-Aldrich, St. Louis, MO, USA), as per the instruction manual. For first-strand cDNA synthesis, 1 μg of total RNA was converted to cDNA using iScript cDNA Synthesis Kit following the manufacturer’s instructions (Bio-Rad Laboratories, USA). The primer sequences used for the qRT-PCR analysis of *OsHyPRP* genes are listed in Supplementary Table S2. The qRT-PCR reactions were performed in 20 µL reaction mixture using 2x iQ SYBR Green Supermix (Bio-Rad, California, USA) with universal cycling conditions (95 °C; 5 min, 40 cycles of 95 °C for 10 s and 60 °C for 60 s) and melt curve analysis on a CFX96 Touch Real-Time PCR detection system (Bio-Rad). Each reaction was performed with three technical replicates of each independent biological replicates.

### 3.6. Promoter Sequence Analysis of Cis-Regulatory Elements

The 1.5 kb region 5′ upstream of the start codon of selected *OsHyPRP* genes was retrieved from RAP-DB (rapdb.dna.affrc.go.jp/) and analyzed using the PLACE web server (www.hsls.pitt.edu/obrc/index.php?page=URL1100876009) to find the potential *cis*-acting regulatory elements.

### 3.7. Protein Structure Prediction and Docking Analysis

The 3D Structures of the candidate protein was predicted through I-Tasser software and further refined by ModRefiner [79,80]. The Ramachandran plot for predicted protein structure was analyzed using Rampage software (mordred.bioc.cam.ac.uk/~rapper/rampage.php). After validation of protein structure by SAVES server (https://services.mbi.ucla.edu/SAVES/), docking analysis was performed by using molecular modeling simulation software, AutoDock 4, available at Docking Server, as 100 iterations with default parameters [81,82]. For protein-ligand docking analysis, the structure of lipid, sterols and cell wall components was retrieved from PubChem (pubchem.ncbi.nlm.nih.gov) and converted to Docking Server compatible pdb format using babel tool [83].

## 4. Conclusions

In the current study, we systematically identified 45 *OsHyPRP* genes and characterized them for their chromosomal distribution, gene structure, phylogenetic relationship, duplication analysis, and expression profiling under different experimental conditions. The *OsHyPRP* genes are mostly intron-less with biased chromosomal distribution and are categorized into seven clades based on their phylogenetic relationship. We also identified some new candidate *OsHyPRP* genes that were not identified previously. Furthermore, the qRT-PCR based expression profiling revealed the early induced up-regulated expression of *OsHyPRP16* under stress conditions and hormone treatment. Additionally, the mature OsHyPRP16 protein was predicted to exhibit a strong binding with various lipids, lipid derivatives, fungal cell-wall components, and phytohormones based on the *in silico* modeling. All these results enable us to put forward the *OsHyPRP16* as a promising candidate for future functional analysis that could help to devise strategies for the development of a rice cultivar tolerant to multiple stresses. However, owing to the rapid sequence diversification through ongoing gene duplication in due course of evolution, the functional diversification or possible neofunctionalization of *HyPRPs* in rice can never be overruled.

## Figures and Tables

**Table 1:** List of Hybrid proline-rich proteins (HyPRPs) in rice and some of their features.

Locus IDs	Nomenclature	Signal Peptide		Mature Protein		
		Amino Acid	Target ^a^	Amino Acid	Mass (kDa)	pI
LOC_Os02g44310.1	OsHyPRP1	22	S	111	11.27	8.14
LOC_Os02g44320.1	OsHyPRP2	22	S	104	10.65	6.24
LOC_Os02g49280.1	OsHyPRP3	28	S	233	23.6	5.43
LOC_Os03g01300.1	OsHyPRP4	29	S	110	11.32	5.04
LOC_Os03g01320.1	OsHyPRP5	24	S	160	13.97	8.37
LOC_Os03g14615.1	OsHyPRP6	25	S	404	41.07	8.91
LOC_Os03g14630.1	OsHyPRP7	25	S	103	10.66	8.50
LOC_Os03g14642.1	OsHyPRP8	26	S	122	12.29	8.15
LOC_Os03g14654.1	OsHyPRP9	26	S	132	13.1	8.44
LOC_Os03g26800.1	OsHyPRP10	27	S	112	11.16	6.20
LOC_Os03g43050.1	OsHyPRP11	20	S	221	23	4.69
LOC_Os04g46810.1	OsHyPRP12	23	S	105	10.8	8.65
LOC_Os04g46820.1	OsHyPRP13	23	S	106	10.91	8.43
LOC_Os04g46830.1	OsHyPRP14	22	S	115	7.4	8.02
LOC_Os04g52250.1	OsHyPRP15	27	S	175	17.56	4.88
LOC_Os04g52260.1	OsHyPRP16	32	S	122	12.18	8.04
LOC_Os06g07220.1	OsHyPRP17	22	S	224	23.35	8.96
LOC_Os06g43600.1	OsHyPRP18	27	S	228	23.89	8.86
LOC_Os06g43600.2	OsHyPRP19	27	S	228	23.89	8.86
LOC_Os06g46780.1	OsHyPRP20	28	S	240	23.79	5.51
LOC_Os06g46870.1	OsHyPRP21	28	S	242	23.98	5.51
LOC_Os07g29230.1	OsHyPRP22	26	S	247	25.26	5.24
LOC_Os07g37385.1	OsHyPRP23	26	S	283	28.86	9.89
LOC_Os07g39640.1	OsHyPRP24	19	M	186	19.14	4.72
LOC_Os07g43290.2	OsHyPRP25	22	S	141	14.61	8.40
LOC_Os10g09920.1	OsHyPRP26	25	S	103	10.36	3.97
LOC_Os10g11370.1	OsHyPRP27	21	S	336	35.39	8.88
LOC_Os10g11730.1	OsHyPRP28	23	S	143	15.18	5.15
LOC_Os10g20830.1	OsHyPRP29	23	S	114	11.75	6.44
LOC_Os10g20840.1	OsHyPRP30	23	S	114	11.82	6.86
LOC_Os10g20860.1	OsHyPRP31	23	S	110	11.51	5.98
LOC_Os10g20890.1	OsHyPRP32	28	S	98	10.17	7.08
LOC_Os10g40420.1	OsHyPRP33	34	S	128	12.58	8.68
LOC_Os10g40430.1	OsHyPRP34	23	S	123	12.27	7.07
LOC_Os10g40440.1	OsHyPRP35	23	S	119	12.05	6.62
LOC_Os10g40460.1	OsHyPRP36	25	S	106	10.96	6.85
LOC_Os10g40470.1	OsHyPRP37	24	S	107	10.99	6.92
LOC_Os10g40480.1	OsHyPRP38	24	S	112	11.58	7.72
LOC_Os10g40510.1	OsHyPRP39	21	S	112	11.49	7.38
LOC_Os10g40520.1	OsHyPRP40	22	S	100	10.42	8.00
LOC_Os10g40530.1	OsHyPRP41	21	S	111	11.55	8.05
LOC_Os10g40614.1	OsHyPRP42	26	S	141	13.17	7.91
LOC_Os11g02165.1	OsHyPRP43	29	C	222	22.95	9.45
LOC_Os12g02105.1	OsHyPRP44	28	C	219	22.43	9.35
LOC_Os12g28880.1	OsHyPRP45	24	C	148	15.28	8.74
LOC_Os12g29040.1	OsHyPRP46	20	M	190	20.01	8.26

^a^ Subcellular target for each protein. S: secretory pathway; M: mitochondria: C: chloroplast.

**Table 2:** The predicted molecular docking energy level and other features for the interaction of various ligands with mature and fragment deleted OsHyPRP16.

	Ligand	Estimated Free Energy of Binding (kcal/mol)	vdW + Hbond + Desolvation Energy (kcal/mol)	Electrostatic Energy (kcal/mol)	Total Intermolecular Energy (kcal/mol)	Frequency (%)	Interaction Surface
**Mature OsHyPRP16**							
Sterols	Lanosterol	−5.65	−7.37	−0.01	−7.38	75	869.90
	Desmosterol	−8.88	−10.61	−0.04	−10.65	67	809.07
	Cholesterol	−9.02	−10.68	−0.01	−10.69	57	817.93
	Fungisterol	−7.28	−9.42	−0.08	−9.5	23	819.20
	Ergosterol	−8.08	−9.52	−0.01	−9.53	64	832.67
Lipids and plant hormones	Linolenic acid	−6.36	−9.15	−0.32	−9.48	12	677.53
	JA	−6.09	−6.41	−0.9	−7.31	90	526.62
	MeJA	−5.56	−6.91	−0.01	−6.92	33	540.28
	SA	−5.54	−5.66	−0.11	−5.77	21	484.46
Pathogen cell wall components	NAG	−5.5	−5.64	−0.11	−5.75	21	484.33
	NAM	−6.31	−6.33	−0.44	−6.77	48	609.37
**Fragment Deleted OsHyPRP16**							
Sterols	Lanosterol	0.7	−0.96	−0.01	−0.97	50	867.84
	Desmosterol	4.86	1.84	−0.05	−4.89	30	616.49
	Cholesterol	8.4	−3.13	−0.02	−315%	40	722.06
	Fungisterol	2.7	−4.84	−0.05	−4.89	30	716.49
	Ergosterol	4.08	1.26	0.06	1.32	40	743.26
Lipids and plant hormones	Linolenic acid	2.2	−0.95	−0.46	−1.42	1	600.68
	JA	−5.35	−6.31	−0.64	−6.95	70	538.91
	MeJA	−2.63	−4.28	−0.04	−4.32	24	447.63
	SA	−2.3	−2.78	−0.13	−2.91	19	339.79
Pathogen cell wall components	NAG	−2.38	−2.72	−0.05	−2.76	15	381.84
	NAM	−2.21	−3.1	−0.22	−3.32	4	422.58

## Supplementary Materials

The following are available online at https://www.mdpi.com/2223-7747/8/9/343/s1, Figure S1: Collinear genes pairs of *OsHyPRP* genes on all 12 rice chromosomes. Red lines indicate segmental duplication of *OsHyPRP* genes on all 12 rice chromosomes and gray lines represent the collinear segments other than of *OsHyPRP* genes; Figure S2: Tandem and collinear pairs of duplicated *OsHyPRP* genes in rice genome as predicted through MCScanX; Figure S3: The intron-exon structure of *OsHyPRP* genes. The genomic coordinates of *OsHyPRPs* were used to draw the gene structure. The majority of genes were found to be intron-less; Table S1: List of *cis*-regulatory elements, their sequence, functions and number of elements identified in the 1.5 kb promoter region of four *OsHyPRP* genes; Table S2: The sequence of the primers used for qRT-PCR based expression analysis.
