# Supplementary material for: Genome-Wide Analysis and Expression Profiling of Rice Hybrid Proline-Rich Proteins in Response to Biotic and Abiotic Stresses, and Hormone Treatment
Source: Plants (Basel). 2019 Sep 11;8(9):343. doi: 10.3390/plants8090343 (PMC6784160; doi:10.3390/plants8090343)
Supplement: Supplementary file 1 [file plants-08-00343-s001.zip › Supplementary_files/Table S2.docx]

**Table S2.** The sequence of the primers used for qRT-PCR based expression analysis.

| **Primer name** | **Sequence** | **Amplicon length** |
| --- | --- | --- |
| OsHyPRP5_Fwd | GGCTTCCAGTGCAGCAACTAATA |  |
| OsHyPRP5_Rev | TGGTGGTCGTTCTTCGTACGT | 87bp |
| OsHyPRP14_Fwd | TCGTCAACATCCCGGTGAA |  |
| OsHyPRP14_Rev | CAAGTTTGTTTAAGCGCAGGTGTA | 89bp |
| OsHyPRP15_Fwd | CATCAAGGCCAAGGCGCTC |  |
| OsHyPRP15_Rev | ATATGTACGGTGGATCAGTGGAT | 125bp |
| OsHyPRP16_Fwd | GTCATGGAGATGTGGAGGCA |  |
| OsHyPRP16_Rev | CATATATGCATGACACAGCAAA | 102bp |
| OsHyPRP39_Fwd | TCACATTGGGACACTGCAG |  |
| OsHyPRP39_Rev | CAGTTTCCAAAGTTCAGAGAG | 122bp |
| OsHyPRP40_Fwd | CCTCAGCCTCATCCTCAACAA |  |
| OsHyPRP40_Rev | CAAGCTCGCAGCCATGGT | 110bp |
| OsEF-1a_Fwd | TGGTATGGTGGTGACCTTTG |  |
| OsEF-1a_Rev | GTACCCACGCTTCAGATCCT | 151bp |

Fwd: forward primer; Rev: reverse primer
