# Supplementary material for: Genome-Wide Analysis and Expression Profiling of Rice Hybrid Proline-Rich Proteins in Response to Biotic and Abiotic Stresses, and Hormone Treatment
Source: Plants (Basel). 2019 Sep 11;8(9):343. doi: 10.3390/plants8090343 (PMC6784160; doi:10.3390/plants8090343)
Supplement: Supplementary file 1 [file plants-08-00343-s001.zip › Supplementary_files/Table S1.docx]

**Table S1.** List of *cis*-regulatory elements, their sequence, functions and number of elements identified in the 1.5Kb promoter region of four *OsHyPRP* genes.

| ***cis*-element** | **Sequence** | **Number of *cis*-elements** | | | | **Functions** |
| --- | --- | --- | --- | --- | --- | --- |
|  | | *OsHyPRP5* | *OsHyPRP14* | *OsHyPRP16* | *OsHyPRP40* |  |
| MARTBOX | TTWTWTTWTT | 2 | 4 | 3 | - | ABA and drought responsive element |
| MYB1AT | WAACCA | 4 | 1 | 1 | 1 | ABA and drought responsive element |
| SBOXATRBCS | CACCTCCA | - | 1 | - | - | ABA and sugar responsive element |
| ACGTABREMOTIFA2OSEM | ACGTGKC | - | - | 1 | - | ABA responsive element |
| DOFCOREZM | AAAG | 6 | 15 | 21 | 17 | ABA responsive |
| DPBFCOREDCDC3 | ACACNNG | - | 1 | - | 3 | ABA responsive |
| RYREPEATBNNAPA | CATGCA | 6 | - | 9 | 3 | ABA responsive |
| LTRECOREATCOR15 | CCGAC | - | - | 2 | - | ABA, drought and cold responsive |
| MYCATRD22 | CACATG | - | 1 | 1 | 2 | ABA, drought and cold responsive |
| MYCCONSENSUSAT | CANNTG | 12 | 6 | 10 | 16 | ABA, drought and cold responsive |
| ABRELATERD1 | ACGTG | 3 | - | 3 | 2 | ABRE-like sequence |
| ASF1MOTIFCAMV | TGACG | - | - | 1 | 1 | Auxin responsive |
| BIHD1OS | TGTCA | 2 | 5 | 6 | 3 | Disease resistance response |
| CBFHV | RYCGAC | 1 | 1 | 3 | 1 | Drought responsive |
| ELRECOREPCRP1 | TTGACC | 1 | 1 | - | 1 | Elicitor responsive |
| WBBOXPCWRKY1 | TTTGACY | 2 | 1 | 1 | - | Elicitor responsive |
| CAREOSREP1 | CAACTC | 1 | - | 1 | 3 | GA responsive |
| EECCRCAH1 | GANTTNC | 1 | - | 1 | 1 | GA responsive |
| GADOWNAT | ACGTGTC | - | - | 1 | - | GA responsive |
| GARE1OSREP1 | TAACAGA | - | - | 1 | - | GA responsive |
| MYBCOREATCYCB1 | AACGG | - | - | 1 | 1 | GA responsive |
| MYBGAHV | TAACAAA | - | 1 | 1 | - | GA responsive |
| PYRIMIDINEBOXOSRAMY1A | CCTTTT | - | - | 3 | 1 | GA responsive |
| CCAATBOX1 | CCAAT | 5 | 3 | 3 | 1 | Heat stress responsive |
| QARBNEXTA | AACGTGT | - | - | 1 | - | JA responsive |
| T/GBOXATPIN2 | AACGTG | - | - | 2 | - | JA responsive |
| GATABOX | GATA | 13 | 13 | 11 | 7 | Light regulated tissue specific expression |
| WBOXATNPR1 | TTGAC | 3 | 3 | 6 | 5 | Pathogen and SA responsive |
| GT1GMSCAM4 | GAAAAA | 2 | 1 | 1 | 6 | Pathogen and salt responsive |
| TATCCAOSAMY | TATCCA | - | 1 | - | 1 | Pathogen and salt responsive |
| WRKY71OS | TGAC | 7 | 10 | 14 | 5 | Pathogen responsive |
| ACGTATERD1 | ACGT | 10 | 4 | 10 | 4 | SA responsive |
| EBOXBNNAPA | CANNTG | 12 | 6 | 10 | 16 | SA responsive |
| GT1CONSENSUS | GRWAAW | 8 | 8 | 9 | 17 | SA responsive |
| MYB1LEPR | GTTAGTT | 1 | - | - | - | Stress responsive |
| WBOXNTERF3 | TGACY | 5 | 5 | 7 | 1 | Wounding responsive |
