# Supplementary figures and images for: Genome-Wide Analysis and Expression Profiling of Rice Hybrid Proline-Rich Proteins in Response to Biotic and Abiotic Stresses, and Hormone Treatment
Source: Plants (Basel). 2019 Sep 11;8(9):343. doi: 10.3390/plants8090343 (PMC6784160; doi:10.3390/plants8090343)

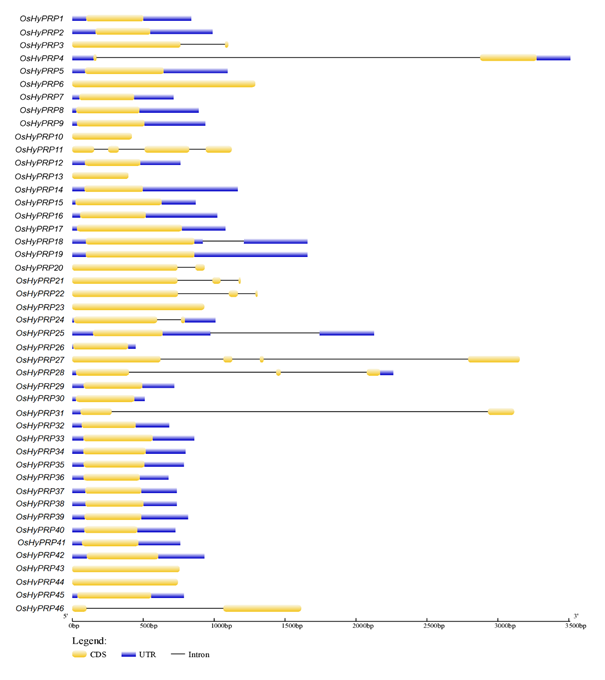

Supplement: Supplementary file 1 [file plants-08-00343-s001.zip › Supplementary_files/Figure S3.png]

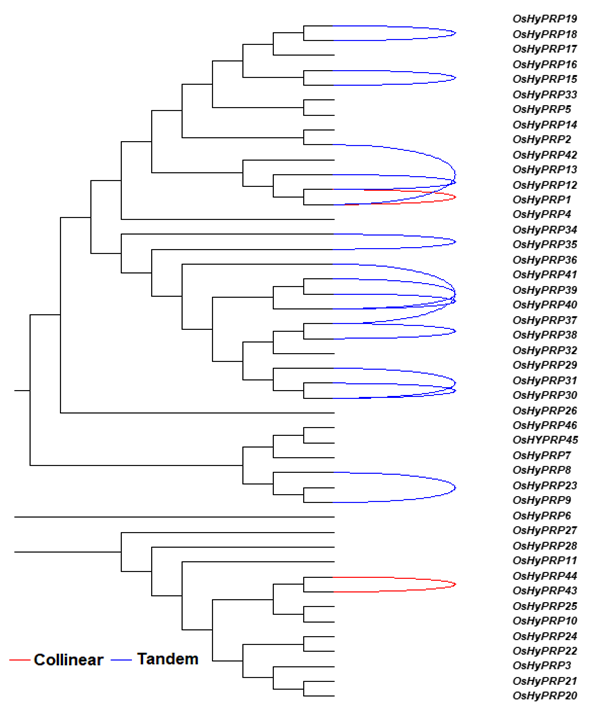

Supplement: Supplementary file 1 [file plants-08-00343-s001.zip › Supplementary_files/Figure S2.png]

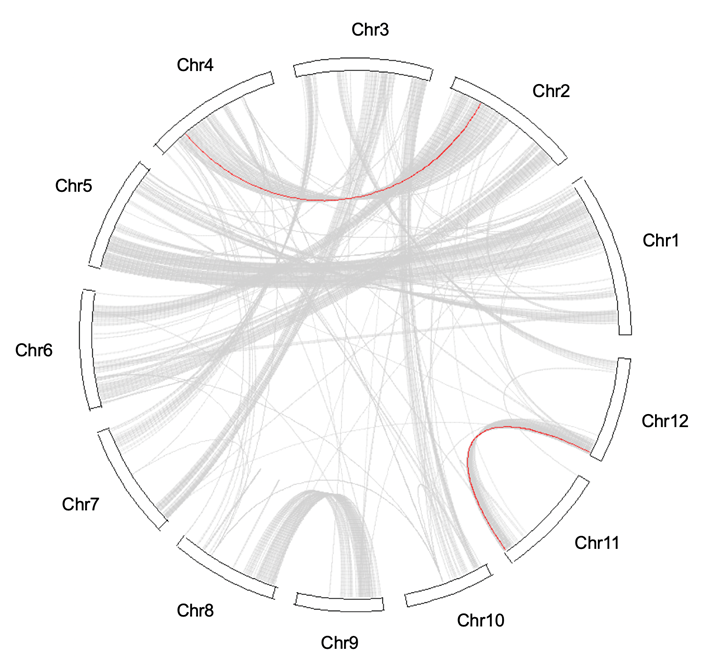

Supplement: Supplementary file 1 [file plants-08-00343-s001.zip › Supplementary_files/Figure S1.png]
